# Characterization of Knowledge, Attitudes, and Practices on Bovine Mastitis, Bacteriological Culture, and Antimicrobial Use on Tropical Dairy Farms

**DOI:** 10.1155/vmi/3663657

**Published:** 2026-09-16

**Authors:** Giovanny Torres-Lindarte, Julián Reyes-Velez, Mónica Jiménez-Serna, Arley Caraballo-Guzmán

**Affiliations:** ^1^ Colombian Institute of Tropical Medicine-CES University, Sabaneta, Antioquia, Colombia

## Abstract

Antimicrobial resistance (AMR) is a growing global concern, strongly linked to the misuse of antimicrobials in livestock production. In dairy farming, producers, milkers, and veterinarians play key roles in antimicrobial decision‐making. This study aimed to characterize knowledge, attitudes, and practices (KAP) related to bovine mastitis, bacteriological culture, and antimicrobial use on tropical dairy farms in Colombia and to explore their association with sociodemographic factors. A total of 366 farm decision‐makers were surveyed across five municipalities in Antioquia. 56.6% of respondents had never used microbiological culture for mastitis diagnosis, and only 41.3% kept records of antimicrobial use. Most participants (98.6%) considered antimicrobials easy to obtain, while only 34.3% routinely consulted veterinarians before use. Blanket dry cow therapy was common practice (81.2%). KAP scores were positively correlated, with higher values associated with larger herd size and greater farming experience, whereas lower educational attainment and reliance on manual milking were linked to poorer scores. Although awareness of AMR was high (88.0%), half of the respondents (50.5%) did not perceive it as a threat to their farms. These findings highlight persistent gaps in antimicrobial stewardship and diagnostic use, underscoring the need for targeted educational interventions, improved access to on‐farm diagnostic tools, and strengthened regulatory frameworks to promote sustainable antimicrobial use in tropical dairy systems.

## 1. Introduction

Bovine mastitis represents a major challenge for the dairy industry worldwide, resulting in substantial economic losses and potential adverse effects on both animal and human health [1]. Current estimates suggest that nearly 70% of global antimicrobial consumption occurs in the agricultural sector, primarily for livestock production and disease prevention [2]. For instance, a study conducted in Dutch dairy farms reported that around 73% of antimicrobials are administered to treat and prevent intramammary infections (IMI), and almost two‐thirds of these are applied during dry cow therapy [3].

The unnecessary use of these drugs in animal production can lead to the development of antimicrobial resistance (AMR), which the World Health Organization (WHO) has identified as one of the top 10 global public health threats [4]. Global antimicrobial consumption is projected to increase by 8% by 2030, reaching 107,472 tons, compared to the 99,502 tons reported in 2020. South America is expected to be one of the regions with the highest growth in antimicrobial use (14%), surpassed only by Oceania (16%) and Africa (25%) [2].

In dairy farming, producers, milkers, and veterinarians are key stakeholders in antimicrobial use. Reducing usage requires improving both their knowledge and behavior [5]. The strategies implemented to manage IMI and other aspects of udder health largely depend on how the problem is perceived. Decision‐making about antimicrobial use to treat IMI in dairy herds is deeply influenced by what stakeholders know and believe, and how they act on it [6, 7]. Consequently, intervention approaches vary widely, often leading to suboptimal practices, including the misuse and overuse of antimicrobials, as documented in several studies [8, 9]. In addition, the lack of use of diagnostic tools such as microbiological culture, along with the absence of surveillance programs, regulations on antimicrobial use, stewardship plans, and effective measures for the control and prevention of IMI, limits the appropriate use of antimicrobials on farms [10].

The knowledge, attitudes, and practices (KAP) model is commonly used to investigate health‐related issues through the assessment of current knowledge, attitudes, and practices about specific health issues through structured surveys [11]. Designed by expert staff to collect information about a specific topic, the KAP model relies on standardized and validated tools to ensure the validity and reliability of the information obtained, which is subsequently analyzed [11].

The KAP approach offers valuable tools for understanding these dynamics and can support efforts to improve antimicrobial stewardship on farms. Enhancing knowledge and awareness among key stakeholders regarding antimicrobial use may represent a cost‐effective strategy, particularly in countries where antimicrobials are often used without veterinary prescription. Lack of information is a significant barrier to developing effective strategies to optimize antimicrobial use and promote more sustainable practices [12]. However, the identification of determinants influencing dairy stakeholders’ knowledge, attitudes, and practices concerning antibiotic use remains insufficiently explored [5], specifically in dairy tropical systems. Dairy production systems in tropical regions exhibit distinct structural and management characteristics that may directly influence antimicrobial use. These systems are typically based on grazing, with varying degrees of intensification ranging from extensive to semi‐intensive, and share similarities with production systems described in other regions, such as European smallholder farms [13]. In general, tropical dairy systems are characterized by smaller herd sizes and limited access to veterinary services and diagnostic tools. Therefore, the aims of this study were to characterize KAP on bovine mastitis, bacteriological culture, and antimicrobial use on tropical dairy farms and explore the association between sociodemographic characteristics and KAP scores.

## 2. Materials and Methods

### 2.1. Study Design and Population

A descriptive observational study was conducted between August 1 and December 31, 2023. A survey on KAP was applied to 366 decision‐makers: owners (57.1%) and administrators and/or managers of dairy farms (42.9%) in the municipalities of San Pedro de los Milagros, Entrerríos, Santa Rosa de Osos, La Ceja, and Rionegro in the department of Antioquia.

### 2.2. Study Design and Questionnaire Development

Using the Google Forms platform, a digital KAP survey was designed. The initial design process included a participatory construction exercise involving professionals in microbiology and veterinary medicine with extensive technical and scientific knowledge on the subject. Through a brainstorming‐type consultation, a first set of 47 questions was generated.

This set of questions was reviewed, adjusting it according to the previously defined categories of analysis. Questions containing key elements identified during the literature review were added. Subsequently, this version of the KAP survey was reviewed for content by the project’s principal investigator, who provided the necessary feedback.

The adjusted version of the survey was sent to a group of four experts in bacteriology, microbiology, and animal sciences, who conducted a detailed review and suggested technical and form adjustments. The new version of the KAP questionnaire was shared with a group of four veterinarians, experts in the subject of the study. These experts reviewed and completed the instrument, making specific clarifications, but without making significant changes.

As a result of this content validation exercise, which included a detailed and critical review by technical experts, a final instrument composed of 71 questions was obtained (Supporting Information 1). These questions were grouped into four components structured according to the predefined categories of analysis: sociodemographic variables, knowledge, attitudes, and practices regarding the diagnosis of bovine mastitis, knowledge, attitudes, and practices regarding the management of information and knowledge, attitudes, and practices regarding the use of antimicrobials, and were ready to be submitted to the next validation step: the pilot test. The next step was the empirical validation to test the reliability of the research instrument called pilot test, which consisted of selecting a subgroup of participants with the same characteristics as the participants of the established sample and applying the instrument to them.

A pilot test was conducted with 36 volunteers (10% of the target population) who met the same inclusion criteria as the full sample. The questionnaire was administered under identical conditions, with responses monitored daily. Pilot data were analyzed to refine the instrument prior to its application to the full study sample.

The questionnaire’s reliability was assessed through a pilot application to decision‐makers, confirming clear comprehension of all items. The instrument was deemed feasible, requiring no more than 30 min to complete, and was aligned with its intended objectives. The pilot results were methodological rather than diagnostic, with emphasis placed on evaluating the technical soundness of the instrument. This process enabled refinement and improvement of the questionnaire, strengthening its methodological rigor.

The final, validated, and piloted version of the KAP questionnaire was approved for implementation and internally circulated among the research team and project experts to ensure familiarity prior to field application. The questionnaire was then made available online to a predefined sample of 366 decision‐makers, including dairy farm owners, administrators, and managers, each of whom received a personalized invitation explaining the study objectives and providing access to the survey. Follow‐up reminders were sent periodically by email or telephone to nonrespondents. Survey completion was continuously monitored through the online platform, with daily verification of data entry, completeness, and quality. Progress was tracked through daily counts and reports to the research team, complemented by weekly summaries assessing response rates and overall advancement.

### 2.3. Ethics Approval

This project was approved by the research ethics board (Minutes No. 77 of the Ethics Committee) of the Colombian Institute of Tropical Medicine (ICMT). All participants were informed about the objectives, methods, and possible implications and/or benefits of the study. In addition, it was ensured that all participants were over 18 years of age and were informed of their rights to freely decide to participate. Each survey participant authorized the processing of data by the ICMT and their consent to participate, registering yes, on the first page of the survey.

### 2.4. Demographic and Herd Production Variables

Demographic variables assessed included geographical location, respondent age, sex, educational background, farm role, milking system type, milking location, and total number of cows in production.

### 2.5. Statistical Analysis

#### 2.5.1. Scoring KAP Questions

Items (71 questions) included in the KAP analysis were limited to single‐response questions to ensure consistent ordinal scoring and comparability across respondents, while demographic variables were excluded from scale construction to avoid conflation of outcome and explanatory variables. Individual item responses were scored using a standardized ordinal system (0 = poor, 0.5 = intermediate, 1 = good), and domain‐specific KAP scores were calculated as the sum of item scores per participant [9]. Moreover, knowledge, attitudes, and practices were conceptualized as latent constructs measured through composite questionnaire scores, and an additive scoring method was selected as a previously reported approach for applied KAP research, using item response theory [14].

Three different categorical variables based on the total KAP scores were calculated, using the percentile distribution: “good,” “moderate,” or “poor,” using the ≥ 75th percentile, < 75th to 25th percentile, and < 25th percentile of the individual scores [6]. The categorical KAP scores were used as ordinal variables to explore the association between sociodemographic variables and each specific section score.

#### 2.5.2. Descriptive Analysis

Descriptive statistics were utilized to summarize the variables of interest, and the distribution of KAP scores was examined across each question block.

#### 2.5.3. Correlations Between Knowledge, Attitudes, Practices

Further correlations among the KAP scores were assessed using the Spearman correlation (Table 1) statistic with a statistical significance of *p* < 0.05.

**TABLE 1:** Spearman correlation coefficients.

**Knowledge**	**Attitude**	**Practices**	

Knowledge	1		
Attitude	0.5130^∗^	1	
Practices	0.7012^∗^	0.4981^∗^	1

^∗^ *p* < 0.05.

#### 2.5.4. Ordinal Logistic Regression

To identify sociodemographic variables associated with the KAP scores, univariate ordinal logistic regression was used. The proportional assumption was assessed for each of the demographic variables, and further examination of the estimates was performed using partial proportional odds univariate model [15–17]. These analyses were carried out using Stata, Version 18.0 (StataCorp LLC, College Station, TX), and R Version 4.0.0 (2020‐04‐24).

## 3. Results

### 3.1. Demographic Variables

These results are presented in Table 2. Most respondents were from Santa Rosa (35.0%) and San Pedro (27.9%), with smaller proportions from Entrerríos (14.8%), Rionegro (13.4%), and La Ceja (9.0%).

**TABLE 2:** Demographic characteristics of dairy herds.

Variable	Frequency	%
Entrerríos	54	14.75
La Ceja	33	9.02
Rionegro	49	13.39
San Pedro	102	27.87
Santa Rosa	128	34.97
Age in years		
< 32	84	22.95
≥ 32 < 52	186	50.82
≥ 52	96	26.23
Sex		
Female	75	20.49
Male	291	79.51
Education		
Specialization	19	5.19
None	3	0.82
Postgraduate	11	3.01
Primary	64	17.49
Professional	116	31.69
High school	99	27.05
College	22	6.01
Technician	32	8.74
Job in farm		
Administrator	157	42.9
Owner	209	57.1
Years of work experience		
1–5 years	61	16.67
10–15 years	46	12.57
15–20 years	54	14.75
5–10 years	52	14.21
< 1 year	16	4.37
> 20 years	137	37.43
Milking type		
Automated	1	0.27
Manual	28	7.65
Manual, mechanic	2	0.55
Mechanic	334	91.26
Mechanic, automated	1	0.27
Place of milking		
Paddock	75	20.49
Paddock Parlour	6	1.64
Parlour	285	77.87
Total cows are production		
1–50	167	45.63
51–100	119	32.51
> 100	80	21.86

The predominant age group was 32–51 years (50.8%), followed by ≥ 52 years (26.2%) and < 32 years (23.0%). The respondents were largely male (79.5%). Educational levels were highest for professional degrees (31.7%) and high school education (27.1%), with fewer reporting primary (17.5%), technical (8.7%), college (6.0%), specialization (5.2%), postgraduate (3.0%), or no formal education (0.8%). Over half were farm owners (57.1%), and 37.4% had more than 20 years of work experience. Mechanical milking systems predominated (91.3%), with most milking conducted in parlors (77.9%). Herd sizes were typically small to medium, with 45.6% managing 1–50 cows, 32.5% managing 51%–100%, and 21.9% managing more than 100 cows in production.

### 3.2. Dairy Farmers’ KAP

**FIGURE 1:**
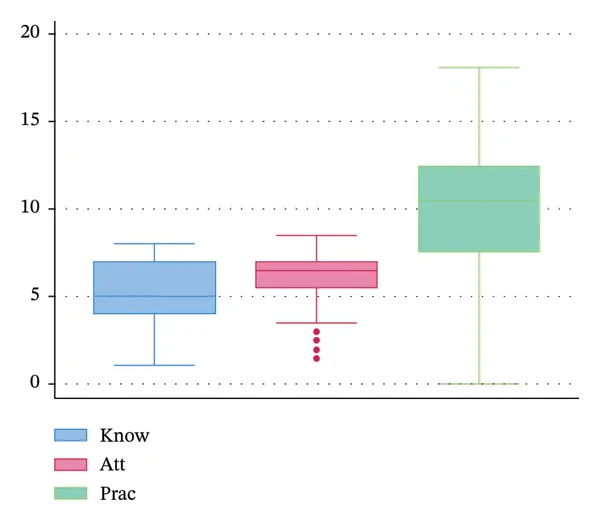
Score distribution of knowledge, attitudes, and practices on bovine mastitis, bacteriological culture, and antimicrobial use in dairy farms from Antioquia Colombia. Know: knowledge; Att: attitudes; Prac: practices. Score axis based on “good,” “moderate,” or “poor” using the ≥ 75th percentile, < 75th to 25th percentile, and < 25th percentile of the individual scores.

The distribution of the KAP scores was presented in Figure 1 and the frequency distribution by parameters is shown in Tables 3–5. For the knowledge parameters block, almost all respondents had heard of bovine mastitis (99.5%) and could correctly define it (99.7%). When facing difficulty interpreting microbiological culture results, 59.8% relied on external assistance, while 40.2% were able to interpret them independently. The bacteria most frequently reported as causing mastitis on farms were identified correctly by 22.7% of respondents, with 64.2% selecting an incorrect option and 13.1% partially correct. Regarding the most difficult bacteria to treat, 46.7% identified the correct pathogen, 50.8% selected incorrectly, and 2.5% were partially correct. Nearly all participants (97.5%) reported having knowledge of antibiotics; however, only 45.1% were aware that they act exclusively against bacteria, and 44.8% had received training on their appropriate use. Awareness of AMR in mastitis pathogens was reported by 88.0% of respondents.

**TABLE 3:** Knowledge parameters on bovine mastitis, bacteriological culture, and antimicrobial use in dairy farms from Antioquia Colombia.

Knowledge parameters	KAP scores		
	0	0.5	1
Have you ever heard of bovine mastitis?			
Freq.	2		364
Percent	0.55		99.45
What is bovine mastitis?			
Freq.	1		363
Percent	0.27		99.73
If you have difficulty understanding the microbiological culture results, who helps you interpret them?			
Freq.	219		147
Percent	59.84		40.16
Of the following bacteria, which one generates the most cases of mastitis on your farm?			
Freq.	235	48	83
Percent	64.21	13.11	22.68
Of the following bacteria, which one do you consider to be the most difficult to treat?			
Freq.	186	9	171
Percent	50.82	2.46	46.72
Do you know what for antimicrobials are used for?			
Freq.	9		357
Percent	2.46		97.54
Have you attended any courses/training on the use of antimicrobials?			
Freq.	202		164
Percent	55.19		44.81
Have you heard about the antimicrobial resistance that bacteria that cause mastitis can present?			
Freq.	44		322
Percent	12.02		87.98

**TABLE 4:** Attitudes parameters on bovine mastitis, bacteriological culture, and antimicrobial use in dairy farms from Antioquia Colombia.

Attitude parameters	KAP scores		
	0	0.5	1
If a cow has a swollen udder, redness of the teats and pain on palpation, changes in the appearance of the milk, such as: red or yellow color and lumps, would you say that cow has			
Freq.	14		352
Percent	3.83		96.17
If you do not see any changes in the udder or milk, but have a positive CMT greater than (+) or a high somatic cell count (SCC) (greater than 200,000 cells/mL), would you say that animal has?			
Freq.	18		348
Percent	4.92		95.08
When you decide to perform a microbiological culture, where do you send the samples?			
Freq.	239	78	49
Percent	65.3	21.31	13.39
Would you like to have the information of the results of the microbiological culture, somatic cell count (SCC dairy control) and CMT on your cell phone?			
Freq.	18		348
Percent	4.92		95.08
Are the results easy to understand?			
Freq.	302	60	4
Percent	82.51	16.39	1.09
If you had the opportunity to perform microbiological cultures yourself and on your own farm to find out which bacteria are causing mastitis and to be able to treat them quickly, would you be willing to use it?			
Freq.	17		349
Percent	4.64		95.36
How much would you be willing to pay for a microbiological culture that you can do yourself on the farm?			
Freq.	47	87	232
Percent	12.84	23.77	63.39
Do you think it is easy to acquire antimicrobials?			
Freq.	361		5
Percent	98.63		1.37
Do you think you are using antimicrobials correctly?			
Freq.	12	110	244
Percent	3.28	30.05	66.67
Do you think antimicrobial resistance is a problem for your farm?			
Freq.	185		181
Percent	50.55		49.45

**TABLE 5:** Practices parameters on bovine mastitis, bacteriological culture, and antimicrobial use in dairy farms from Antioquia Colombia.

Practice parameters	KAP score		
	0	0.5	1
Do you use or have you used microbiological culture to detect or treat mastitis?			
Freq.	207		159
Percent	56.56		43.44
In which cases do you use microbiological culture?			
Freq.	208	56	102
Percent	56.83	15.3	27.87
What do you do with the milk samples after collection?			
Freq.	304	48	14
Percent	83.06	13.11	3.83
After collecting the milk sample, how long does it take to send it to the laboratory?			
Freq.	242	69	55
Percent	66.12	18.85	15.03
How long does it take to get the results of the microbiological culture?			
Freq.	228	83	55
Percent	62.3	22.68	15.03
How do you store the results of Somatic Cell Count (RCS dairy control) and CMT (palleting) tests?			
Freq.	138	136	92
Percent	37.7	37.16	25.14
How do you store microbiological culture results?			
Freq.	187	114	65
Percent	51.09	31.15	17.76
Does the tool you use to store or analyze Somatic Cell Count (SCR milk control), CMT (palleting) or microbiological culture results belong to a supplier of inputs and services?			
Freq.	268		98
Percent	73.22		26.78
What is the diagnostic test that you use the most to make antimicrobial treatment decisions?			
Freq.	72	240	44
Percent	20.22	67.42	12.36
Do you have a written protocol that guides you on which antimicrobial to use?			
Freq.	267		99
Percent	72.95		27.05
Who is the person who commonly applies the antimicrobial treatments on the farm?			
Freq.	10	340	16
Percent	2.73	92.9	4.37
Where do you buy the antimicrobials?			
Freq.	10	350	6
Percent	2.73	95.63	1.64
How much (in Colombian pesos) have you spent on antimicrobials on your dairy farm in the last 12 months?			
Freq.	107	123	136
Percent	29.23	33.61	37.16
When you are going to use an antimicrobial, do you seek advice from a veterinarian?			
Freq	50	194	122
Percent	13.66	53.01	33.33
Do you record the use of antimicrobials in animals?			
Freq.	149	70	147
Percent	40.71	19.13	40.16
How do you currently record the use of veterinary antimicrobials on your farm?			
Freq.	140	80	146
Percent	38.25	21.86	39.89
How do you store antimicrobials?			
Freq.	46	264	56
Percent	12.57	72.13	15.3
What do you do with leftover antimicrobials?			
Freq.	207	80	79
Percent	56.56	21.86	21.58
Does having antimicrobials available on the farm make the decision to use them easier?			
Freq.	355		11
Percent	96.99		3.01
Do you strictly follow the antimicrobial manufacturer’s recommendations for use?			
Freq.	16	85	265
Percent	4.37	23.22	72.4
What measures do you apply to reduce the use of antimicrobials on the farm?			
Freq.	30	336	
Percent	8.2	91.8	
What measures do you apply when an animal does not respond to the antimicrobial administered?			
Freq.	149	207	10
Percent	40.71	56.56	2.73
How long do you wait to apply another antimicrobial treatment?			
Freq.	36	182	148
Percent	9.84	49.73	40.44
Do you apply antimicrobials to all animals entering the dry‐off period?			
Freq.	69		297
Percent	18.85		81.15

On the Attitudes toward mastitis diagnosis, testing, and antimicrobial use block, most of the respondents (≥ 95%) correctly recognized both clinical and subclinical mastitis scenarios, expressed willingness to receive diagnostic information on their mobile phones, and indicated they would use on‐farm microbiological culture if available. Most (82.5%) found the current test results easy to understand. However, 65.3% sent culture samples to external laboratories, with smaller proportions using regional or private facilities. The majority (63.4%) were willing to pay the highest proposed amount for on‐farm culture. Almost all (98.6%) considered antimicrobials easy to obtain, and two‐thirds (66.7%) believed they used them correctly. Perceptions of AMR as a problem were evenly distributed (50.6% vs. 49.5%).

For the Practice parameters block, less than half of respondents (43.4%) reported using microbiological culture for mastitis diagnosis, and most did not follow optimal sample handling or timely submission practices. Written antimicrobial treatment protocols were uncommon (27.1%), and record‐keeping for antimicrobial use was inconsistent. While nearly all farms stored antimicrobials appropriately and found them easy to obtain, over half retained leftovers for later use, and blanket dry‐off therapy was widespread (81.2%). Most treatment decisions were based on California Mastitis Test (CMT) or somatic cell count (SCC) results rather than culture, and although 72.4% claimed to follow manufacturer instructions, preventive strategies to reduce antimicrobial use were limited in scope.

### 3.3. Ordinal Univariate Regression

The univariate analysis revealed several significant associations across knowledge, attitudes, and practices (Table 6). In terms of knowledge, respondents from Rionegro (OR = 0.341; *p* = 0.004) and Santa Rosa (OR = 0.387; *p* = 0.002) had significantly lower odds of higher knowledge levels. Lower educational, particularly primary (OR = 0.109; *p* < 0.001) and high school (OR = 0.195; *p* = 0.001) education was strongly associated with reduced knowledge, while more years of farming experience, especially 10–20 years: 10–15 years (OR = 2.492; *p* = 0.005); > 15–20 years (OR = 3.363; *p* < 0.001), and larger herd sizes: 51–100 cows (OR = 2.655; *p* < 0.001); > 100 cows (OR = 4.161; *p* < 0.001) were positively associated with higher knowledge. Manual milking (OR = 0.123; *p* < 0.001) was negatively associated with knowledge. Regarding attitudes, individuals from Rionegro (OR = 0.247; *p* < 0.001) and those with primary (OR = 0.274; *p* = 0.02) or high school (OR = 0.257; *p* = 0.008) education showed significantly lower odds of positive attitudes. Experience between 10 and 15 years (OR = 2.359; *p* = 0.01) and > 15–20 years (OR = 1.962; *p* = 0.04) and owning more than 100 cows (OR = 2.692; *p* < 0.001) were positively associated with better attitudes, while manual milking (OR = 0.231; *p* < 0.001) was again negatively associated. For practices, lower education levels: primary (OR = 0.150; *p* < 0.001) and high school (OR = 0.203; *p* = 0.001) and being a farm owner (OR = 0.600; *p* = 0.01) were linked to poorer practices. Farming experience of 1–20 years (Table 6) and larger herd sizes: 51–100 cows (OR = 3.074; *p* < 0.001) and > 100 cows (OR = 3.896; *p* < 0.001) were positively associated with better practices, while manual milking (OR = 0.078; *p* = 0.001) was significantly associated with poorer practices. These findings highlight the influence of education, experience, and farm characteristics on dairy farmers’ knowledge, attitudes, and practices.

**TABLE 6:** Univariate ordinal regressions for knowledge, attitudes, and practices scores on bovine mastitis, bacteriological culture, and antimicrobial use in dairy farms by and demographic variables from Antioquia Colombia.

Variable	Knowledge					Attitudes					Practices				
	Odds ratio	SE	p > z	CI [95%]		Odds ratio	SE	p > z	CI [95%]		Odds ratio	SE	p > z	CI [95%]	
s_municipality															
La Ceja	0.742	0.299	0.46	0.337	1.633	0.640	0.271	0.29	0.279	1.467	0.616	0.262	0.26	0.268	1.419
Rionegro	0.341	0.127	**0.004**	0.164	0.707	0.247	0.097	**< 0.000**	0.115	0.533	0.695	0.259	0.33	0.335	1.443
San Pedro	0.631	0.198	0.14	0.341	1.167	0.817	0.270	0.54	0.427	1.562	1.539	0.481	0.17	0.834	2.838
Santa Rosa	0.387	0.119	**0.002**	0.212	0.706	0.578	0.183	0.08	0.311	1.074	0.558	0.172	0.06	0.305	1.021
age_cat															
≥ 32 < 52	0.819	0.201	0.42	0.507	1.325	0.772	0.197	0.31	0.468	1.274	0.971	0.239	0.90	0.600	1.572
≥ 52	0.618	0.170	0.08	0.361	1.059	0.607	0.174	0.08	0.346	1.064	0.780	0.224	0.39	0.445	1.368
s_sex															
Masc	0.865	0.204	0.54	0.544	1.374	0.866	0.216	0.56	0.532	1.410	0.757	0.186	0.26	0.468	1.224
s_education															
Postgraduate	3.378	2.549	0.11	0.770	14.824	0.596	0.498	0.54	0.116	3.065	3.160	2.511	0.15	0.666	15.001
Primary	0.109	0.057	**< 0.000**	0.039	0.302	0.274	0.146	**0.02**	0.097	0.777	0.150	0.077	**< 0.000**	0.055	0.412
Professional	1.381	0.657	0.50	0.544	3.506	0.515	0.265	0.20	0.188	1.409	0.935	0.425	0.88	0.383	2.278
High school	0.195	0.095	**0.001**	0.075	0.507	0.257	0.132	**0.008**	0.094	0.704	0.203	0.097	**0.001**	0.080	0.516
College	0.617	0.362	0.41	0.195	1.950	0.404	0.258	0.16	0.116	1.410	0.360	0.214	0.09	0.112	1.154
Technician	1.256	0.702	0.68	0.420	3.758	0.810	0.485	0.73	0.251	2.617	1.123	0.601	0.83	0.394	3.204
s_job_farm															
Owner	0.797	0.156	0.25	0.543	1.171	0.713	0.146	0.10	0.477	1.066	0.600	0.121	**0.01**	0.404	0.891
s_years_exp															
1–5 years	1.925	0.549	**0.02**	1.101	3.368	1.431	0.429	0.23	0.795	2.576	2.510	0.734	**0.002**	1.416	4.452
10–15 years	2.492	0.806	**0.005**	1.321	4.698	2.359	0.796	**0.01**	1.218	4.572	2.088	0.697	**0.03**	1.085	4.019
15–20 years	3.363	1.043	**< 0.000**	1.831	6.176	1.962	0.631	**0.04**	1.044	3.686	3.398	1.062	**< 0.000**	1.841	6.270
5–10 years	1.611	0.491	0.12	0.886	2.928	0.902	0.286	0.75	0.485	1.678	2.065	0.669	**0.03**	1.094	3.897
< 1 year	0.926	0.464	0.88	0.347	2.474	1.052	0.508	0.92	0.408	2.713	1.410	0.718	0.50	0.520	3.826
m_type															
manual	0.123	0.055	**< 0.000**	0.051	0.297	0.231	0.092	**< 0.000**	0.106	0.502	0.078	0.058	**0.001**	0.018	0.335
manual‐mechanic	3.196	4.182	0.37	0.246	41.521	0.740	1.125	0.84	0.038	14.578	1.515	1.553	0.69	0.203	11.302
s_total_cows															
51–100	2.655	0.609	**< 0.000**	1.693	4.163	1.395	0.322	0.15	0.887	2.194	3.074	0.747	**< 0.000**	1.910	4.949
> 100	4.161	1.098	**< 0.000**	2.481	6.978	2.692	0.742	**< 0.000**	1.568	4.620	3.896	1.026	**< 0.000**	2.325	6.529

*Note:* Bold numbers correspond to a *p* < 0.05 significance. Red numbers are estimates that require further assessment due to violation of the proportional assumption**.**

Abbreviations: CI: confidence interval; SE: standard error.

Most of the demographic variables met the odds/parallel lines assumption for each KAP parameter. However, for the Knowledge parameter, the milking type did not meet this assumption, and for the Practices parameter, specific variables such as total cows, years of experience, and job in the farm departed from the parallel regression assumption. When this occurred, the proportionality constraints were imposed using further assessment with a partial proportional odds univariate model for those specific variables (Table 6).

## 4. Discussion

In Colombia, as in many other developing economies, antimicrobials are accessible without a prescription and are often dispensed over the counter, reflecting limited regulatory oversight by public health authorities. This study highlights both the lack of antimicrobial access control and the variability in herd‐level knowledge, practices, and attitudes shaped by production technologies.

A total of 98.6% of respondents reported that antimicrobials were easy to access, and only 34.3% consulted a veterinarian before using them, findings consistent with those reported in other studies [10, 18–20]. Producers commonly seek veterinary advice only after initial treatments fail, as reported by the majority of respondents (58.1%). This behavior has also been documented in earlier studies, where delaying veterinary consultation is perceived as a cost‐saving strategy [10, 20]. Moreover, administering treatments without prior consultation with a professional may lead to improper dosage or course of therapy required to ensure the therapeutic response [10].

Some studies have also highlighted the pressure that veterinarians face from producers to prescribe antimicrobials [20–22]. Notably, 41.6% of participants reported initiating a new treatment immediately after completing the previous one, without observing the recommended interval to confirm bacteriological cure.

The magnitude of the problem is further exacerbated by the fact that only 45.1% of respondents were aware that antimicrobials are effective exclusively against bacterial infections, a misconception also reported in previous studies [18]. This lack of knowledge, combined with limited surveillance and easy access to antimicrobials, significantly increases the risk of misuse, thereby contributing to the emergence and spread of AMR [10, 19].

The growing global pressure to reduce antimicrobial use in livestock production and preserve their efficacy has led several countries, particularly in Europe, to implement measures that restrict their use for preventive purposes (e.g., blanket dry cow therapy) and to strengthen antimicrobial stewardship programs [12, 18, 23–25]. In this study, most participants (72.2%) reported not having a formal protocol for antimicrobial administration on their farms, and only 41.3% maintained any record of antimicrobial use. Furthermore, 96.9% stated that having antimicrobials readily available on the farm facilitated the decision to use them. This finding is especially relevant considering previous studies, which have shown that some veterinarians and producers believe that not using antimicrobials could compromise animal health and welfare, and consequently, productivity [9, 20].

In our study, although most participants had heard of AMR (88.0%), 50.5% did not consider it a relevant issue for their farms. This lack of awareness regarding the contribution of antimicrobial use in livestock to the development of AMR has been previously reported in other studies [18, 26]. The overuse of antimicrobials represents a social dilemma comparable to the “tragedy of the commons,” in which the shared resource being compromised is the effectiveness of these drugs [27]. A study conducted across eight countries, involving approximately 42,000 individuals, revealed that most people tend to prioritize individual benefits, even at the expense of collective welfare [27]. This tendency, combined with limited public awareness of AMR, hinders global control efforts. For instance, in the context of dairy production, it is common practice on some farms to feed calves with milk from treated cows, which may contain antimicrobial residues [10, 28, 29]. This milk is sometimes even sold through informal channels to produce dairy products, often with full awareness of the associated risks [19]. A report by the European Food Safety Authority highlighted the risk of feeding calves with milk containing antimicrobial residues [28]. Continuous exposure to subtherapeutic concentrations of these drugs, present in discarded milk, may promote the excretion and dissemination of antimicrobial‐resistant bacteria [10, 28].

Several studies have shown that increasing awareness about the appropriate use of antimicrobials by enhancing knowledge contributes to improving attitudes and, consequently, promotes good practices regarding this issue [9, 25, 30]. In our study, a positive correlation was identified among the three variables: knowledge, attitudes, and practices. The strongest relationship was observed between knowledge and practice, suggesting that higher levels of knowledge are associated with greater adoption of appropriate practices.

Education level has previously been associated with knowledge levels in earlier reports [9, 30]. In our study, a basic educational background, such as primary (OR = 0.109; *P*<.001) or high school education (OR = 0.195; *P* = .001), was significantly associated with a lower likelihood of exhibiting high levels of knowledge regarding antimicrobial use and AMR, compared to individuals with university level or higher education. Similarly, farms using manual milking methods showed a reduced probability of achieving high knowledge scores (OR = 0.123; *P*<.001). In contrast, work experience emerged as a positive determinant. Workers with 10–15 years of experience (OR = 2.492; *p* = 0.005) and those with 15–20 years (OR = 3.363; *p* < 0.001) had a significantly greater likelihood of possessing higher levels of knowledge. Herd size was also positively associated with knowledge: producers managing 51–100 milking cows (OR = 2.655; *p* < 0.001) or more than 100 cows (OR = 4.161; *p* < 0.001) were more likely to demonstrate high levels of knowledge.

In agreement with previous studies [9, 30, 31], this study also found that lower educational levels were associated with less favorable attitudes toward antimicrobial use and AMR (primary education: OR = 0.274, *p* = 0.02; high school education: OR = 0.257, *p* = 0.008). Likewise, manual milking was related to more negative attitudes (OR = 0.231; *p* < 0.001). In contrast, workers with more than 10 years of experience (10–15 years: OR = 2.359, *p* = 0.01; 15–20 years: OR = 1.962, *p* = 0.04), as well as those working on farms with more than 100 milking cows (OR = 2.692; *p* < 0.001), showed significantly more positive attitudes.

A similar pattern was observed for practices. Worker experience (1–5 years: OR = 2.510, *p* = 0.002; 5–10 years: OR = 2.065, *p* = 0.03; 10–15 years: OR = 2.088, *p* = 0.03; 15–20 years: OR = 3.398, *p* < 0.001) and herd size (51–100 cows: OR = 3.074, *p* < 0.001; more than 100 cows: OR = 3.896, *p* < 0.001) were associated with a higher likelihood of implementing good practices. In contrast, manual milking (OR = 0.078; *p* = 0.001) and lower educational levels (primary: OR = 0.150, *p* < 0.001; high school: OR = 0.203, *p* = 0.001) were associated with poorer practices.

In Colombia, manual milking is still commonly used, particularly on smallholder farms (fewer than 50 milking cows), which are characterized by lower levels of technological development and management routines that increase the risk of pathogen transmission between animals, as well as occupational risk. It is important to note that poor management practices have previously been associated with increased antimicrobial use [32]. In our study, all farms using manual milking had fewer than 50 milking cows; with one exception, all were managed by individuals with only basic education (primary or high school), which may help explain the lower levels of knowledge, attitudes, and practices observed in these farms.

A common issue in therapeutic decision‐making for IMIs is the use of antimicrobials without prior microbiological analysis. Treatment is often initiated without identifying the etiological agent or to treat apparently healthy animals, as occurs with blanket dry cow therapy. A study conducted in Denmark reported that 91% of surveyed veterinarians always or often initiated treatment immediately after clinical evaluation [33]. However, microbiological culture is a key tool in promoting the rational use of antimicrobials, as not all infections require antimicrobial treatment, and some may require extended therapies, as recommended for certain types of infections [34–36]. Several factors, including parity, infection chronicity, and the type of pathogen involved (e.g., *S. aureus* or *S. uberis*), have been associated with a low likelihood of treatment success [37, 38]. In the case of infections caused by Gram‐negative bacteria such as *E. coli*, it has been documented that most cases resolve spontaneously without the need for antimicrobials [39]. Likewise, infections caused by nonculturable pathogens, which do not respond to antimicrobial therapy and yield negative culture results, do not require treatment [40]. In such scenarios, the use of microbiological culture allows for more targeted therapeutic interventions or alternative management strategies (e.g., segregation or culling), thereby avoiding unnecessary treatments and contributing to the mitigation of AMR.

In this study, 56.6% of respondents reported never having used microbiological culture as a tool to guide diagnosis. According to other studies, the most common reasons for not using it include cost, delays in obtaining results, and the lack of alignment between available diagnostic tools and the needs of producers and professionals [10, 20, 41, 42]. A total of 52.2% of participants indicated that culture results take between 4 and 7 days to be delivered. Additionally, a lack of awareness regarding the benefits of this tool for therapeutic decision‐making represents another major barrier to its adoption [18]. The availability of highly sensitive and specific point‐of‐care tests could facilitate their implementation both on farms and in veterinary practice [18, 22, 26].

## 5. Conclusions

The findings of this study highlight multiple challenges to the responsible management of antimicrobials in dairy production, particularly in contexts like Colombia, where conditions that promote inappropriate use persist. Easy access to these antimicrobials and other controlled veterinary products, the absence of formal protocols for their administration, limited knowledge about AMR, and the low rate of veterinary consultation prior to treatment all reflect significant gaps in producer awareness and training.

This study confirms that factors such as educational level, work experience, herd size, and milking system directly influence knowledge, attitudes, and practices related to antimicrobial use. Specifically, manual milking and low levels of education were associated with less appropriate practices, reinforcing the need for tailored strategies targeting these smallholder dairy farmers. Despite general awareness of AMR, many producers still do not perceive it as a direct threat to their farms, which hinders the adoption of preventive measures. Likewise, the use of antimicrobials without prior microbiological diagnosis remains a common practice, even though culture testing enables more targeted interventions and reduces unnecessary treatments.

This context underscores the urgent need to strengthen educational strategies, promote the use of accessible and reliable diagnostic tools, and implement effective policies to regulate access to and use of antimicrobials. Only then will it be possible to move toward more sustainable dairy production aligned with global efforts to contain AMR.

### 5.1. Limitations of the Study

This study had several limitations, including uneven geographical representation of dairy producers across municipalities, the absence of milk quality variables to assess the impact of KAP parameters at the farm level, and the high variability in individual dairy production practices. This presents a potential limitation in identifying independent factors that may influence KAP distribution.

## Funding

This study was funded by “El Sistema General de Regalías – Minciencias,” Colombia (grant number BPIN 2021000100277).

## Conflicts of Interest

The authors declare no conflicts of interest.

## Supporting Information

Additional supporting information can be found online in the Supporting Information section.

## Supporting information


**Supporting Information** Supporting information 1: The file contains the complete questionnaire with the questions and answers obtained from the 366 farms participating in the study.

## Data Availability

The DAS confirms the presence of data. The data used for the current study are available upon request from the corresponding author.
