# Supplementary material for: Characterization of Knowledge, Attitudes, and Practices on Bovine Mastitis, Bacteriological Culture, and Antimicrobial Use on Tropical Dairy Farms
Source: Vet Med Int. 2026 Sep 16;2026:3663657. doi: 10.1155/vmi/3663657 (PMC13581408; doi:10.1155/vmi/3663657)
Supplement: Supplementary file 1 [file VMI-2026-3663657-s001.docx]

**Supplementary Material 1: Questionnaire**

| 1. Municipality where the farm is located (Select one option only) | | |
| --- | --- | --- |
| municipality | n | percent |
| Entrerríos | 54 | 14.8% |
| Santa Rosa | 128 | 35.0% |
| San Pedro | 102 | 27.9% |
| La Ceja | 33 | 9.0% |
| Rionegro | 49 | 13.4% |

| 2. Participant’s age | | |
| --- | --- | --- |
| age | n | percent |
| 20 | 3 | 0.8% |
| 22 | 4 | 1.1% |
| 23 | 5 | 1.4% |
| 24 | 6 | 1.6% |
| 25 | 6 | 1.6% |
| 26 | 5 | 1.4% |
| 27 | 10 | 2.7% |
| 28 | 8 | 2.2% |
| 29 | 12 | 3.3% |
| 30 | 15 | 4.1% |
| 31 | 10 | 2.7% |
| 32 | 17 | 4.6% |
| 33 | 8 | 2.2% |
| 34 | 14 | 3.8% |
| 35 | 7 | 1.9% |
| 36 | 5 | 1.4% |
| 37 | 9 | 2.5% |
| 38 | 9 | 2.5% |
| 39 | 15 | 4.1% |
| 40 | 13 | 3.6% |
| 41 | 7 | 1.9% |
| 42 | 13 | 3.6% |
| 43 | 7 | 1.9% |
| 44 | 12 | 3.3% |
| 45 | 9 | 2.5% |
| 46 | 5 | 1.4% |
| 47 | 10 | 2.7% |
| 48 | 5 | 1.4% |
| 49 | 6 | 1.6% |
| 50 | 7 | 1.9% |
| 51 | 8 | 2.2% |
| 52 | 5 | 1.4% |
| 53 | 4 | 1.1% |
| 54 | 3 | 0.8% |
| 55 | 6 | 1.6% |
| 56 | 6 | 1.6% |
| 57 | 10 | 2.7% |
| 58 | 4 | 1.1% |
| 59 | 4 | 1.1% |
| 60 | 7 | 1.9% |
| 61 | 1 | 0.3% |
| 62 | 5 | 1.4% |
| 63 | 2 | 0.5% |
| 64 | 4 | 1.1% |
| 65 | 4 | 1.1% |
| 66 | 1 | 0.3% |
| 67 | 7 | 1.9% |
| 69 | 2 | 0.5% |
| 70 | 5 | 1.4% |
| 71 | 3 | 0.8% |
| 72 | 1 | 0.3% |
| 74 | 1 | 0.3% |
| 75 | 1 | 0.3% |
| 76 | 4 | 1.1% |
| 78 | 2 | 0.5% |
| 79 | 2 | 0.5% |
| 82 | 1 | 0.3% |
| 83 | 1 | 0.3% |

| 3. Participant’s gender | | |
| --- | --- | --- |
| sex | n | percent |
| Male | 291 | 79.5% |
| Female | 75 | 20.5% |

| 4. Participant's Educational Level (Select one option only) | | |
| --- | --- | --- |
| Education | n | percent |
| None | 3 | 0.8% |
| Primary | 64 | 17.5% |
| High school | 99 | 27.0% |
| Technician | 32 | 8.7% |
| College | 22 | 6.0% |
| Professional | 116 | 31.7% |
| Specialization | 19 | 5.2% |
| Postgraduate | 11 | 3.0% |

| 5. Participant's Role on the Farm (Select one option only) | | |
| --- | --- | --- |
| job_farm | n | percent |
| Owner | 209 | 57.1% |
| Administrator | 157 | 42.9% |

| 6. How many years of experience do you have in this position? (Select only one answer) | | |
| --- | --- | --- |
| years_exp | n | percent |
| < 1 year | 16 | 4.4% |
| 1-5 year | 61 | 16.7% |
| 5-10 year | 52 | 14.2% |
| 10 -15 year | 46 | 12.6% |
| 15-20 year | 54 | 14.8% |
| > 20 year | 137 | 37.4% |

| 7. How many cows does the farm currently have? (Select only one answer) | | |
| --- | --- | --- |
| total_cows | n | percent |
| 1-50 | 167 | 45.6% |
| 51-100 | 119 | 32.5% |
| > 100 | 80 | 21.9% |

| 8. What milking system or technique is used on the farm? (You can select multiple options) | | |
| --- | --- | --- |
| milk_type | n | percent |
| Automated | 1 | 0.3% |
| Manual | 28 | 7.7% |
| Manual, Mechanical | 2 | 0.5% |
| Mechanical | 334 | 91.3% |
| Mechanical, Automated | 1 | 0.3% |

| 9. Where does the milking take place? (You can select multiple options) | | |
| --- | --- | --- |
| place_milk | n | percent |
| Paddock | 75 | 20.5% |
| Paddock, Milking parlor | 6 | 1.6% |
| Milking parlor | 285 | 77.9% |

| 10. Have you heard of bovine mastitis? (Select only one answer) | | |
| --- | --- | --- |
| talk_mast | n | percent |
| Yes | 364 | 99.5% |
| No | 2 | 0.5% |

| 11. What is bovine mastitis? (Select only one answer) | | | |
| --- | --- | --- | --- |
| what_mast | n | percent | valid_percent |
| Correct | 363 | 99.2% | 99.7% |
| Incorrect | 1 | 0.3% | 0.3% |
| NA | 2 | 0.5% | - |

| 12. If a cow has a swollen udder, redness of the teats and pain upon palpation, changes in the appearance of the milk, such as: red or yellow color and clots, would you say that cow has? (Select only one answer) | | | |
| --- | --- | --- | --- |
| mast_diag_self | n | percent | valid_percent |
| Subclinical Mastitis | 37 | 10.1% | 10.2% |
| Clinical Mastitis | 253 | 69.1% | 69.5% |
| All of the above | 60 | 16.4% | 16.5% |
| None | 14 | 3.8% | 3.8% |
| NA | 2 | 0.5% | - |

| 13. If you do not see any change in the udder or the milk, but you have a positive CMT (paddle test) greater than (+) or a high Somatic Cell Count (SCC dairy control) (greater than 200,000 cells/ml), would you say that animal has? (Select only one answer) | | | |
| --- | --- | --- | --- |
| recs_change_diag | n | percent | valid_percent |
| Subclinical Mastitis | 275 | 75.1% | 75.5% |
| Clinical Mastitis | 55 | 15.0% | 15.1% |
| All of the above | 16 | 4.4% | 4.4% |
| None | 18 | 4.9% | 4.9% |
| NA | 2 | 0.5% | - |

| 14. To detect if an animal has mastitis, do you use any of the following tests: CMT, Somatic Cell Count (SCC dairy control) or microbiological culture? (Select only one answer) | | | |
| --- | --- | --- | --- |
| mast_diag_use_farm | n | percent | valid_percent |
| Yes | 308 | 84.2% | 84.6% |
| No | 56 | 15.3% | 15.4% |
| NA | 2 | 0.5% | - |

| 15. Which of the following tests or methods do you use to detect mastitis? (You can select multiple options) | | | |
| --- | --- | --- | --- |
| masti_diag_farm | n | percent | valid_percent |
| CMT | 151 | 41.3% | 49.2% |
| CMT, Culture | 3 | 0.8% | 1.0% |
| CMT, Culture, Visual | 2 | 0.5% | 0.7% |
| CMT, Portable device | 2 | 0.6% | 0.6% |
| CMT, SCC | 40 | 10.9% | 13.0% |
| CMT, SCC, Culture | 22 | 6.0% | 7.2% |
| CMT, SCC, Culture, Portable device | 1 | 0.3% | 0.3% |
| CMT, SCC, Culture, Portable device, Visual | 7 | 1.9% | 2.3% |
| CMT, SCC, Culture, Visual | 13 | 3.6% | 4.2% |
| CMT, SCC, Visual | 22 | 6.0% | 7.1% |
| CMT, Visual | 11 | 3.0% | 3.6% |
| Culture | 3 | 0.8% | 1.0% |
| SCC | 14 | 3.8% | 4.6% |
| SCC, Culture | 1 | 0.3% | 0.3% |
| SCC, Culture, Visual | 1 | 0.3% | 0.3% |
| SCC, Visual | 3 | 0.8% | 1.0% |
| Visual | 11 | 3.0% | 3.6% |
| NA | 59 | 16.1% | - |

| 16. How often do you use the CMT (paddle test) to detect mastitis? (You can select multiple options) | | | |
| --- | --- | --- | --- |
| cmt_freq | n | percent | valid_percent |
| At the time of drying off | 5 | 1.3% | 1.6% |
| Every 15 days | 50 | 13.6% | 16.2% |
| Every 15 days, At the time of drying off | 3 | 0.8% | 0.9% |
| Every 15 days, Only when the cow has mastitis | 1 | 0.3% | 0.3% |
| Every 15 days, Only when the cow has mastitis, At the time of drying off | 1 | 0.3% | 0.3% |
| Every two months | 19 | 5.2% | 6.1% |
| Every two months, At the time of drying off | 2 | 0.5% | 0.6% |
| Every month | 118 | 32.2% | 38.3% |
| Every month, At the time of drying off | 3 | 0.8% | 1.0% |
| Every month, Only when the cow has mastitis | 4 | 1.1% | 1.3% |
| Every month, Only when the cow has mastitis, At the time of drying off | 2 | 0.5% | 0.6% |
| Every six months | 4 | 1.1% | 1.3% |
| Every six months, Only when the cow has mastitis | 2 | 0.6% | 0.6% |
| Every six months, Only when the cow has mastitis, At the time of drying off | 1 | 0.3% | 0.3% |
| None of the above | 18 | 4.9% | 5.8% |
| Weekly | 35 | 9.6% | 11.4% |
| Weekly, At the time of drying off | 4 | 1.1% | 1.3% |
| Weekly, Only when the cow has mastitis | 1 | 0.3% | 0.3% |
| Only when the cow has mastitis | 33 | 9.0% | 10.7% |
| Only when the cow has mastitis, At the time of drying off | 2 | 0.5% | 0.6% |
| NA | 58 | 15.8% | - |

| 17. How often do you use the Somatic Cell Count (SCC dairy control) test to detect mastitis? (You can select multiple options) | | | |
| --- | --- | --- | --- |
| rcs_freq | n | percent | valid_percent |
| At the time of drying off | 1 | 0.3% | 0.3% |
| Every 15 days | 24 | 6.5% | 7.9% |
| Every 15 days, At the time of drying off | 1 | 0.3% | 0.3% |
| Every 15 days, Only when the cow has mastitis | 2 | 0.5% | 0.7% |
| Every two months | 6 | 1.6% | 2.0% |
| Every month | 99 | 27.1% | 32.2% |
| Every six months | 5 | 1.4% | 1.6% |
| Only when the cow has mastitis | 10 | 2.8% | 3.2% |
| None of the above | 133 | 36.3% | 43.3% |
| Weekly | 24 | 6.6% | 7.8% |
| Every month, At the time of drying off | 1 | 0.3% | 0.3% |
| Weekly, Only when the cow has mastitis, At the time of drying off | 1 | 0.3% | 0.3% |
| NA | 59 | 16.1% | - |

| 18. Do you use or have you used microbiological culture to detect or treat mastitis? (Select only one answer) | | | |
| --- | --- | --- | --- |
| cult_use | n | percent | valid_percent |
| Yes | 159 | 43.4% | 51.6% |
| No | 149 | 40.7% | 48.4% |
| NA | 58 | 15.8% | - |

| 19. In which cases do you use the microbiological culture? (You can select multiple options) | | | |
| --- | --- | --- | --- |
| cult_when | n | percent | valid_percent |
| High SCC from milk collector | 1 | 0.3% | 0.6% |
| CMT positive, High SCC from milk collector | 1 | 0.3% | 0.6% |
| CMT positive, High SCC from milk collector, At the time of drying off | 1 | 0.3% | 0.6% |
| CMT positive, No improvement | 2 | 0.5% | 1.3% |
| CMT positive, No improvement, High SCC from milk collector | 2 | 0.6% | 1.2% |
| It is not used | 1 | 0.3% | 0.6% |
| No improvement | 48 | 13.1% | 30.2% |
| No improvement, High SCC from milk collector | 2 | 0.5% | 1.3% |
| High SCC | 7 | 1.9% | 4.4% |
| High SCC, High SCC from milk collector | 3 | 1.3% | 3.2% |
| High SCC, Recurrent animals | 1 | 0.3% | 0.6% |
| High SCC, CMT positive | 2 | 0.5% | 1.3% |
| High SCC, No improvement | 6 | 1.6% | 3.8% |
| Mastitis suspicion | 1 | 0.3% | 0.6% |
| CMT positive | 2 | 0.6% | 1.2% |
| High SCC, CMT positive, High SCC from milk collector | 1 | 0.3% | 0.6% |
| Clinical mastitis, CMT positive, No improvement, High SCC from milk collector | 2 | 0.6% | 1.2% |
| Clinical mastitis, High SCC, High SCC from milk collector | 1 | 0.3% | 0.6% |
| Clinical mastitis | 9 | 2.5% | 5.7% |
| Clinical mastitis, CMT positive | 2 | 0.5% | 1.3% |
| Clinical mastitis, CMT positive, No improvement | 2 | 0.5% | 1.3% |
| Clinical mastitis, No improvement | 14 | 3.8% | 8.8% |
| Clinical mastitis, No improvement, High SCC from milk collector | 2 | 0.5% | 1.3% |
| Clinical mastitis, High SCC | 1 | 0.3% | 0.6% |
| Clinical mastitis, High SCC, No improvement | 2 | 0.5% | 1.3% |
| Clinical mastitis, High SCC, No improvement, High SCC from milk collector | 1 | 0.3% | 0.6% |
| Subclinical mastitis | 4 | 1.1% | 2.5% |
| Subclinical mastitis, CMT positive | 1 | 0.3% | 0.6% |
| Subclinical mastitis, Clinical mastitis, High SCC, No improvement, High SCC from milk collector | 1 | 0.3% | 0.6% |
| Subclinical mastitis, No improvement | 3 | 0.8% | 1.9% |
| Subclinical mastitis, High SCC | 3 | 0.8% | 1.9% |
| Subclinical mastitis, High SCC, CMT positive | 3 | 0.8% | 1.9% |
| Subclinical mastitis, Clinical mastitis, High SCC, No improvement, CMT positive | 1 | 0.3% | 0.6% |
| Subclinical mastitis, Clinical mastitis, High SCC | 1 | 0.3% | 0.6% |
| Subclinical mastitis, Clinical mastitis, Suspicion mastitis, High SCC, CMT positive, No improvement, High SCC from milk collector | 2 | 0.6% | 1.2% |
| Subclinical mastitis, Suspicion mastitis, High SCC | 1 | 0.3% | 0.6% |
| Subclinical mastitis, Clinical mastitis, Suspicion mastitis, CMT positive, No improvement | 1 | 0.3% | 0.6% |
| Subclinical mastitis, Clinical mastitis, Suspicion mastitis, High SCC, No improvement, High SCC from milk collector | 1 | 0.3% | 0.6% |
| Subclinical mastitis, Clinical mastitis | 1 | 0.3% | 0.6% |
| Subclinical mastitis, Clinical mastitis, No improvement | 6 | 1.6% | 3.8% |
| Subclinical mastitis, Clinical mastitis, High SCC | 1 | 0.3% | 0.6% |
| Subclinical mastitis, Clinical mastitis, High SCC alto, CMT positive | 1 | 0.3% | 0.6% |
| Subclinical mastitis, Clinical mastitis, High SCC, CMT positive, No improvement | 3 | 0.8% | 1.9% |
| Subclinical mastitis, Clinical mastitis, High SCC, No improvement, High SCC from milk collector | 1 | 0.3% | 0.6% |
| Subclinical mastitis, Clinical mastitis, High SCC, No improvement | 5 | 1.4% | 3.1% |
| NA | 207 | 56.6% | - |

| 20. What difficulties do you encounter when performing the culture? (You can select multiple options) | | | |
| --- | --- | --- | --- |
| diff_cult | n | percent | valid_percent |
| Sending samples to a lab is very complicated | 29 | 7.9% | 18.2% |
| Sending samples to a lab is very complicated, Results interpretation | 4 | 1.1% | 2.5% |
| Sending samples to a lab is very complicated, Sample collection | 6 | 1.6% | 3.8% |
| Sending samples to a lab is very complicated, Doesn't know a lab to perform the analysis | 1 | 0.3% | 0.6% |
| Results interpretation | 3 | 0.8% | 1.9% |
| Sample collection | 8 | 2.2% | 5.0% |
| Does not have the tools or materials to perform it on the farm | 3 | 0.8% | 1.9% |
| Does not have the tools or materials to perform it on the farm, Sending samples to a lab is very complicated | 7 | 1.9% | 4.4% |
| Does not have the tools or materials to perform it on the farm, Sending samples to a lab is very complicated, Results interpretation | 1 | 0.3% | 0.6% |
| Does not have the tools or materials to perform it on the farm, Sending samples to a lab is very complicated, Sample collection | 1 | 0.3% | 0.6% |
| Does not have the tools or materials to perform it on the farm, Sending samples to a lab is very complicated, Doesn't know a lab to perform the analysis | 1 | 0.3% | 0.6% |
| Does not have the tools or materials to perform it on the farm, Sending samples to a lab is very complicated, Doesn't know what to do to perform a culture, Results interpretation | 1 | 0.3% | 0.6% |
| Does not have the tools or materials to perform it on the farm, Doesn't know what to do to perform a culture | 1 | 0.3% | 0.6% |
| Does not have the tools or materials to perform it on the farm, They are very expensive | 1 | 0.3% | 0.6% |
| Does not have the tools or materials to perform it on the farm, Sending samples to a lab is very complicated, They are very expensive | 2 | 0.5% | 1.3% |
| Does not have the tools or materials to perform it on the farm, Sending samples to a lab is very complicated, They are very expensive, Doesn't know a lab to perform the analysis, Doesn't know what to do to perform a culture, Results interpretation, Sample collection | 1 | 0.3% | 0.6% |
| Does not have the tools or materials to perform it on the farm, Sending samples to a lab is very complicated, They are very expensive, Doesn't know what to do to perform a culture, Results interpretation | 1 | 0.3% | 0.6% |
| Does not have the tools or materials to perform it on the farm, They are very expensive, Sample collection | 1 | 0.3% | 0.6% |
| Finds no difficulties | 70 | 19.1% | 44.0% |
| They are very expensive | 8 | 2.2% | 5.0% |
| They are very expensive, Sending samples to a lab is very complicated | 6 | 1.6% | 3.8% |
| They are very expensive, Sending samples to a lab is very complicated, Results interpretation | 1 | 0.3% | 0.6% |
| They are very expensive, Sending samples to a lab is very complicated, Sample collection | 1 | 0.3% | 0.6% |
| They are very expensive, Results interpretation | 1 | 0.3% | 0.6% |
| NA | 207 | 56.6% | - |

| 21. What do you do with the result sent to you from the culture? (You can select multiple options) | | | |
| --- | --- | --- | --- |
| cases_cult | n | percent | valid_percent |
| Shows the result to the veterinarian to start treatment | 49 | 13.4% | 30.8% |
| Shows the result to the veterinarian to start treatment, Treats the cow according to the result | 28 | 7.7% | 17.6% |
| Other | 25 | 6.8% | 15.7% |
| Shows the result to the veterinarian to start treatment, Treats the cow according to the result, Uses it to modify or correct the milking routine | 14 | 3.8% | 8.8% |
| Treats the cow according to the result | 43 | 11.7% | 27.0% |
| NA | 207 | 56.6% | - |

| 22. When you decide to perform a microbiological culture, where do you send the samples? (Select only one answer) | | | |
| --- | --- | --- | --- |
| where_cult | n | percent | valid_percent |
| Local lab | 67 | 18.3% | 42.1% |
| Medellín lab | 49 | 13.4% | 30.8% |
| Milk collector lab | 32 | 8.7% | 20.1% |
| Veterinarian | 11 | 3.0% | 6.9% |
| NA | 207 | 56.6% | - |

| 23. How do you send the milk sample for microbiological culture? (Select only one answer) | | | |
| --- | --- | --- | --- |
| env_sample_cult | n | percent | valid_percent |
| Sterile container | 102 | 27.9% | 64.2% |
| Urine container | 56 | 15.3% | 35.2% |
| Milk container | 1 | 0.3% | 0.6% |
| NA | 207 | 56.6% | - |

| 24. With whom do you send the milk sample for microbiological culture? (You can select multiple options) | | | |
| --- | --- | --- | --- |
| who_sample_cult | n | percent | valid_percent |
| With the tank truck driver | 35 | 9.6% | 22.0% |
| With the tank truck driver, Veterinarian | 1 | 0.3% | 0.6% |
| With the tank truck driver, Veterinarian, Takes it personally | 1 | 0.3% | 0.6% |
| With the tank truck driver, Takes it personally | 8 | 2.2% | 5.0% |
| Veterinarian | 17 | 4.6% | 10.7% |
| Veterinarian, Courier | 2 | 0.5% | 1.3% |
| Veterinarian, Takes it personally | 4 | 1.1% | 2.5% |
| Courier | 22 | 6.0% | 13.8% |
| Courier, Takes it personally | 6 | 1.6% | 3.8% |
| Takes it personally | 63 | 17.2% | 39.6% |
| NA | 207 | 56.6% | - |

| 25. What do you do with the milk samples after collecting them? (Select only one answer) | | | |
| --- | --- | --- | --- |
| what_cult_samp | n | percent | valid_percent |
| Tank truck driver | 97 | 26.5% | 61.0% |
| Veterinarian | 48 | 13.1% | 30.2% |
| Courier | 14 | 3.8% | 8.8% |
| NA | 207 | 56.6% | - |

| 26. After the milk sample is collected, how long does it take to send it to the lab? (Select only one answer) | | | |
| --- | --- | --- | --- |
| long_env_samp | n | percent | valid_percent |
| 1-2 h | 55 | 15.0% | 34.6% |
| 2-4 h | 45 | 12.3% | 28.3% |
| 4-6 h | 24 | 6.6% | 15.1% |
| 6-12 h | 24 | 6.6% | 15.1% |
| > 12 h | 11 | 3.0% | 6.9% |
| NA | 207 | 56.6% | - |

| 27. How much do you pay for the microbiological culture? (Select only one answer) | | | |
| --- | --- | --- | --- |
| cost_cult | n | percent | valid_percent |
| $15.000-$25.000 COP | 69 | 18.9% | 43.4% |
| $26.000-$35.000 COP | 52 | 14.2% | 32.7% |
| $36.000-$50.000 COP | 25 | 6.8% | 15.7% |
| > $50.000 COP | 13 | 3.6% | 8.2% |
| NA | 207 | 56.6% | - |

| 28. How long does it take to receive the results of the microbiological culture? (Select only one answer) | | | |
| --- | --- | --- | --- |
| how_long_result_samp | n | percent | valid_percent |
| 1-3 days | 55 | 15.0% | 34.6% |
| 4-7 d days | 83 | 22.7% | 52.2% |
| 8-14 days | 19 | 5.2% | 11.9% |
| > 15 days | 2 | 0.5% | 1.3% |
| NA | 207 | 56.6% | - |

| 29. Are the results easy to understand? (Select only one answer) | | | |
| --- | --- | --- | --- |
| easy_results_samp | n | percent | valid_percent |
| Always | 95 | 26.0% | 59.7% |
| Almost always | 45 | 12.3% | 28.3% |
| Sometimes | 15 | 4.1% | 9.4% |
| Never | 4 | 1.1% | 2.5% |
| NA | 207 | 56.6% | - |

| 30. If you have difficulty understanding the results of the microbiological culture, who helps you interpret them? (Select only one answer) | | | |
| --- | --- | --- | --- |
| who_interp_cult | n | percent | valid_percent |
| Professional | 147 | 40.2% | 92.5% |
| Friend or neighbor | 2 | 0.5% | 1.3% |
| Own interpretation | 8 | 2.2% | 5.0% |
| Internet | 2 | 0.5% | 1.3% |
| NA | 207 | 56.6% | - |

| 31. Of the following bacteria, which one causes the most cases of mastitis on your farm? (Select only one answer) | | | |
| --- | --- | --- | --- |
| freq_micro | n | percent | valid_percent |
| S. aureus | 50 | 13.7% | 31.4% |
| S. agalactiae | 33 | 9.0% | 20.8% |
| S. uberis | 20 | 5.5% | 12.6% |
| S. aureus, SNA | 6 | 1.6% | 3.8% |
| E. coli | 22 | 6.0% | 13.8% |
| Does not know | 28 | 7.7% | 17.6% |
| NA | 207 | 56.6% | - |

| 32. Of the following bacteria, which one do you consider the most difficult to treat? (Select only one answer) | | |
| --- | --- | --- |
| diffi_treat_micro | n | percent |
| S. aureus | 154 | 42.1% |
| S. agalactiae | 9 | 2.5% |
| S. uberis | 11 | 3.0% |
| S. aureus, SNA | 6 | 1.6% |
| E. coli | 69 | 18.9% |
| Does not know | 117 | 32.0% |

| 33. If you had the opportunity to perform microbiological cultures yourself on your own farm to find out which bacteria are causing mastitis and treat them quickly; would you be willing to use it? (Select only one answer) | | |
| --- | --- | --- |
| self_cult_if | n | percent |
| Yes | 349 | 95.4% |
| No | 17 | 4.6% |

| 34. How much would you be willing to pay for a microbiological culture that you can perform yourself on the farm? (Select only one answer) | | |
| --- | --- | --- |
| dis_cost_cult | n | percent |
| $15.000-$25.000 COP | 232 | 63.4% |
| $26.000-$35.000 COP | 87 | 23.8% |
| $36.000-$50.000 COP | 30 | 8.2% |
| > $50.000 COP | 17 | 4.6% |

| 35. How do you store the results of Somatic Cell Count (SCC dairy control) and CMT (paddle test)? (Select only one answer) | | |
| --- | --- | --- |
| save_results | n | percent |
| Paper | 92 | 25.1% |
| Electronic | 136 | 37.2% |
| Does not store | 88 | 24.0% |
| Does not perform tests | 50 | 13.7% |

| 36. How do you store the results of microbiological cultures? (Select only one answer) | | |
| --- | --- | --- |
| how_alm_result | n | percent |
| Paper | 65 | 17.8% |
| Electronic | 114 | 31.1% |
| Does not store | 59 | 16.1% |
| Does not perform culture | 128 | 35.0% |

| 37. Does the tool you use to store or analyze the results of Somatic Cell Count (SCC dairy control), CMT (paddle test) or microbiological culture belong to a supplier of inputs and services? (Select only one answer) | | |
| --- | --- | --- |
| tool_alm_result | n | percent |
| Yes | 98 | 26.8% |
| No | 268 | 73.2% |

| 38. For decision-making on your farm, do you consider the results of Somatic Cell Count (SCC dairy control), CMT (paddle test) or microbiological culture? (You can select multiple options) | | |
| --- | --- | --- |
| deci_results | n | percent |
| Does not take them into account | 47 | 12.8% |
| Does not know what to do with the results | 3 | 0.8% |
| Yes, culls problem animals | 3 | 0.8% |
| Yes, culls problem animals, Yes, treats animals with mastitis | 23 | 6.3% |
| Yes, separates infected animals or those with mastitis | 16 | 4.4% |
| Yes, separates infected animals or those with mastitis, Yes, culls problem animals | 4 | 1.1% |
| Yes, separates infected animals or those with mastitis, Yes, culls problem animals, Yes, treats animals with mastitis | 40 | 10.9% |
| Yes, separates infected animals or those with mastitis, Yes, treats animals with mastitis | 38 | 10.4% |
| Yes, treats animals with mastitis | 191 | 52.2% |
| Yes, treats animals with mastitis, Does not take them into account | 1 | 0.3% |

| 39. Would you like to be able to have the information from the microbiological culture, Somatic Cell Count (SCC dairy control) and CMT (paddle test) results on your cell phone? (Select only one answer) | | |
| --- | --- | --- |
| phone_results | n | percent |
| Yes | 348 | 95.1% |
| No | 18 | 4.9% |

| 40. Do you know what antibiotics are used for? (Select only one answer) | | |
| --- | --- | --- |
| abs_what_for | n | percent |
| Yes | 357 | 97.5% |
| No | 9 | 2.5% |

| 41. Antibiotics are used to treat: (You can select multiple options) | | | |
| --- | --- | --- | --- |
| abs_tx | n | percent | valid_percent |
| Bacteria | 161 | 44.0% | 45.1% |
| Bacteria, Fungi | 3 | 0.8% | 0.8% |
| Bacteria, Fungi, Parasites | 4 | 1.1% | 1.1% |
| Bacteria, Parasites | 4 | 1.1% | 1.1% |
| Fungi | 1 | 0.3% | 0.3% |
| Fungi, Parasites | 1 | 0.3% | 0.3% |
| None | 1 | 0.3% | 0.3% |
| Parasites | 1 | 0.3% | 0.3% |
| All | 158 | 43.2% | 44.3% |
| Viruses | 5 | 1.4% | 1.4% |
| Viruses, Bacteria | 12 | 3.3% | 3.4% |
| Viruses, Bacteria, Fungi | 5 | 1.4% | 1.4% |
| Viruses, Bacteria, Fungi | 1 | 0.3% | 0.3% |
| NA | 9 | 2.5% | - |

| 42. Do you use antibiotics on your farm to treat mastitis? (Select only one answer) | | |
| --- | --- | --- |
| abs_use | n | percent |
| Yes | 356 | 97.3% |
| No | 10 | 2.7% |

| 43. In which cases do you use antibiotics? (You can select multiple options) | | | |
| --- | --- | --- | --- |
| cases_abs | n | percent | valid_percent |
| At drying off | 2 | 0.5% | 0.6% |
| When you know it's sick from a microorganism | 23 | 6.3% | 6.5% |
| When you know it's sick from a microorganism, At drying off | 10 | 2.7% | 2.8% |
| When a cow is sick, When the veterinarian tells you | 18 | 4.9% | 5.1% |
| When a cow is sick, When you know it's sick from a microorganism, When the veterinarian tells you, At drying off | 35 | 9.6% | 9.8% |
| When the veterinarian tells you | 34 | 9.3% | 9.6% |
| When the veterinarian tells you, At drying off | 5 | 1.4% | 1.4% |
| When a cow is sick | 123 | 33.6% | 34.6% |
| When a cow is sick, At drying off | 25 | 6.9% | 7.0% |
| When a cow is sick, When you know it's sick from a microorganism | 11 | 3.0% | 3.1% |
| When a cow is sick, When you know it's sick from a microorganism, At drying off | 4 | 1.1% | 1.1% |
| When a cow is sick, When you know it's sick from a microorganism, When the veterinarian tells you | 11 | 3.0% | 3.1% |
| When a cow is sick, When the veterinarian tells you | 46 | 12.6% | 12.9% |
| When a cow is sick, When the veterinarian tells you, At drying off | 9 | 2.5% | 2.5% |
| NA | 10 | 2.7% | - |

| 44. What is the diagnostic test you use most to make antibiotic treatment decisions? (Select only one answer) | | | |
| --- | --- | --- | --- |
| dx_test_use | n | percent | valid_percent |
| CMT | 192 | 52.5% | 53.9% |
| SCC | 48 | 13.1% | 13.5% |
| Culture | 44 | 12.0% | 12.4% |
| No test | 72 | 19.7% | 20.2% |
| NA | 10 | 2.7% | - |

| 45. Do you have a written protocol to guide you on which antibiotic to use? (Select only one answer) | | | |
| --- | --- | --- | --- |
| protocol_written | n | percent | valid_percent |
| Yes | 99 | 27.0% | 27.8% |
| No | 257 | 70.2% | 72.2% |
| NA | 10 | 2.7% | - |

| 46. Who is the person who commonly applies antibiotic treatments on the farm? (Select only one answer) | | | |
| --- | --- | --- | --- |
| who_tx | n | percent | valid_percent |
| The milker | 138 | 37.7% | 38.8% |
| The foreman | 150 | 41.0% | 42.1% |
| The farm owner | 52 | 14.2% | 14.6% |
| The veterinarian | 16 | 4.4% | 4.5% |
| NA | 10 | 2.7% | - |

| 47. In which cases do you NOT use antibiotics? (You can select multiple options) | | | |
| --- | --- | --- | --- |
| cases_no_use_abs | n | percent | valid_percent |
| When the microbiological culture is negative | 107 | 29.2% | 30.1% |
| When the culture result is a Gram-negative bacillus | 8 | 2.2% | 2.2% |
| When the antibiotic withdrawal time is greater than 72 hours | 20 | 5.5% | 5.6% |
| When the antibiotic withdrawal time is greater than 72 hours, When there are bad product recommendations | 9 | 2.5% | 2.5% |
| When there are bad product recommendations | 144 | 39.3% | 40.4% |
| When there are bad product recommendations, When the microbiological culture is negative | 43 | 11.7% | 12.1% |
| When you have a cow in milking and the antibiotic has withdrawal | 11 | 3.0% | 3.1% |
| Other | 14 | 3.8% | 3.9% |
| NA | 10 | 2.7% | - |

| 48. Where do you buy the antibiotics? (Select only one answer) | | | |
| --- | --- | --- | --- |
| where_purch_abs | n | percent | valid_percent |
| Agriculture store | 350 | 95.6% | 98.3% |
| Veterinarian | 6 | 1.6% | 1.7% |
| NA | 10 | 2.7% | - |

| 49. How much (in Colombian pesos) have you spent on antibiotics for your dairy farm in the last 12 months? (Select only one answer) | | | |
| --- | --- | --- | --- |
| cost_tx_abs | n | percent | valid_percent |
| < $500.000 COP | 68 | 18.6% | 20.9% |
| $500.000-$1.000.000 COP | 70 | 19.1% | 21.5% |
| $1.000.000-$2.000.000 COP | 68 | 18.6% | 20.9% |
| $2.000.000-$4.000.000 COP | 53 | 14.5% | 16.3% |
| > $4.000.000 COP | 66 | 18.0% | 20.3% |
| NA | 41 | 11.2% | - |

| 50. Do you think it is easy to acquire antibiotics? (Select only one answer) | | | |
| --- | --- | --- | --- |
| easy_abs | n | percent | valid_percent |
| Yes | 351 | 95.9% | 98.6% |
| No | 5 | 1.4% | 1.4% |
| NA | 10 | 2.7% | - |

| 51. When you are going to use an antibiotic, do you seek advice from a veterinarian? (Select only one answer) | | | |
| --- | --- | --- | --- |
| vet_abs | n | percent | valid_percent |
| Always | 122 | 33.3% | 34.3% |
| Almost always | 66 | 18.0% | 18.5% |
| Sometimes | 128 | 35.0% | 36.0% |
| Almost never | 26 | 7.1% | 7.3% |
| Never | 14 | 3.8% | 3.9% |
| NA | 10 | 2.7% | - |

| 52. Do you record the use of antibiotics in animals? (Select only one answer) | | | |
| --- | --- | --- | --- |
| reg_abs | n | percent | valid_percent |
| Always | 147 | 40.2% | 41.3% |
| Almost always | 38 | 10.4% | 10.7% |
| Sometimes | 32 | 8.7% | 9.0% |
| Almost never | 16 | 4.4% | 4.5% |
| Never | 123 | 33.6% | 34.6% |
| NA | 10 | 2.7% | - |

| 53. How do you currently record the use of veterinary antibiotics on your farm? (Select only one answer) | | | |
| --- | --- | --- | --- |
| reg_how_abs | n | percent | valid_percent |
| Paper | 146 | 39.9% | 62.7% |
| Spreadsheet | 35 | 9.6% | 15.0% |
| Software | 45 | 12.3% | 19.3% |
| Data storage memory | 7 | 1.9% | 3.0% |
| NA | 133 | 36.3% | - |

| 54. How do you store antibiotics? (Select only one answer) | | | |
| --- | --- | --- | --- |
| how_alm_abs | n | percent | valid_percent |
| Refrigerator | 3 | 0.8% | 0.8% |
| With other medications | 261 | 71.3% | 73.7% |
| With all other farm supplies | 34 | 9.3% | 9.6% |
| Only with other antibiotics | 56 | 15.3% | 15.8% |
| NA | 12 | 3.3% | - |

| 55. What do you do with leftover antibiotic? (Select only one answer) | | | |
| --- | --- | --- | --- |
| surp_abs | n | percent | valid_percent |
| Discard it | 79 | 21.6% | 22.2% |
| Use it later with other cows | 196 | 53.6% | 55.1% |
| Give it away | 1 | 0.3% | 0.3% |
| Store it in the refrigerator | 80 | 21.9% | 22.5% |
| NA | 10 | 2.7% | - |

| 56. Does having antibiotics available on the farm facilitate the decision to use them? (Select only one answer) | | | |
| --- | --- | --- | --- |
| abs_avail | n | percent | valid_percent |
| Yes | 345 | 94.3% | 96.9% |
| No | 11 | 3.0% | 3.1% |
| NA | 10 | 2.7% | - |

| 57. Do you strictly follow the manufacturer's usage recommendations for the antibiotic? (Select only one answer) | | | |
| --- | --- | --- | --- |
| rec_abs_comp | n | percent | valid_percent |
| Always | 265 | 72.4% | 74.4% |
| Almost always | 66 | 18.0% | 18.5% |
| Sometimes | 19 | 5.2% | 5.3% |
| Almost never | 6 | 1.6% | 1.7% |
| NA | 10 | 2.7% | - |

| 58. Do you believe you are using antibiotics correctly? (Select only one answer) | | | |
| --- | --- | --- | --- |
| correct_use_abs | n | percent | valid_percent |
| Always | 217 | 59.3% | 61.0% |
| Almost always | 110 | 30.1% | 30.9% |
| Sometimes | 27 | 7.4% | 7.6% |
| Almost never | 1 | 0.3% | 0.3% |
| Never | 1 | 0.3% | 0.3% |
| NA | 10 | 2.7% | - |

| 59. In case the previous answer was never, why do you think you are not? (Select only one answer) | | | |
| --- | --- | --- | --- |
| why_not_correct | n | percent | valid_percent |
| Does not know | 1 | 0.3% | 100.0% |
| NA | 365 | 99.7% | - |

| 60. What measures do you apply to reduce the use of antibiotics on the farm? (Select only one answer) | | | |
| --- | --- | --- | --- |
| red_use_abs | n | percent | valid_percent |
| Infected animals | 152 | 41.5% | 42.7% |
| Those indicated by the veterinarian | 58 | 15.8% | 16.3% |
| Infected animals reaching drying off | 17 | 4.6% | 4.8% |
| Infected animals with the support of tests | 109 | 29.8% | 30.6% |
| None | 20 | 5.5% | 5.6% |
| NA | 10 | 2.7% | - |

| 61. What measures do you apply when an animal does not respond to the administered antibiotic? (Select only one answer) | | | |
| --- | --- | --- | --- |
| no_response_abs | n | percent | valid_percent |
| Use another antibiotic | 139 | 38.0% | 39.0% |
| Call the veterinarian | 207 | 56.6% | 58.1% |
| Cull the cow | 10 | 2.7% | 2.8% |
| NA | 10 | 2.7% | - |

| 62. How long do you wait to apply another antibiotic treatment? (Select only one answer) | | | |
| --- | --- | --- | --- |
| time_ab_other | n | percent | valid_percent |
| Immediately | 148 | 40.4% | 41.6% |
| 7 days | 182 | 49.7% | 51.1% |
| 7-15 days | 26 | 7.1% | 7.3% |
| NA | 10 | 2.7% | - |

| 63. Do you apply antibiotics to all animals entering the drying off period? (Select only one answer) | | | |
| --- | --- | --- | --- |
| dry_cow_ther | n | percent | valid_percent |
| Yes | 297 | 81.1% | 83.4% |
| No | 59 | 16.1% | 16.6% |
| NA | 10 | 2.7% | - |

| 64. In case the previous answer was NO, do you use another type of product? which one? (Select only one answer) | | | |
| --- | --- | --- | --- |
| other_not_dry | n | percent | valid_percent |
| Yes | 37 | 10.1% | 72.5% |
| No | 14 | 3.8% | 27.5% |
| NA | 315 | 86.1% | - |

| 65. When do you treat cows with subclinical mastitis? (You can select multiple options) | | | |
| --- | --- | --- | --- |
| when_mast_sc | n | percent | valid_percent |
| At the beginning of the drying off period | 54 | 14.8% | 15.2% |
| At the beginning of the drying off period, During the lactation period | 61 | 16.7% | 17.1% |
| At the beginning of the drying off period, During the lactation period, Do not treat them | 1 | 0.3% | 0.3% |
| During the lactation period | 183 | 50.0% | 51.4% |
| Do not treat them | 57 | 15.6% | 16.0% |
| NA | 10 | 2.7% | - |

| 66. Which of the following antibiotics do you use most frequently or have you used for drying off animals? (Select only one answer) | | | |
| --- | --- | --- | --- |
| abs_which | n | percent | valid_percent |
| Benzetacil | 1 | 0.3% | 0.3% |
| Calimast | 1 | 0.3% | 0.3% |
| Calimax Secado | 2 | 0.5% | 0.6% |
| Calimax, Secador | 1 | 0.3% | 0.3% |
| Ceftiofur Sodico | 1 | 0.3% | 0.3% |
| Cepravin | 173 | 47.3% | 48.6% |
| Ciprolac | 23 | 6.3% | 6.5% |
| Cloxacum | 2 | 0.5% | 0.6% |
| Ganapen | 1 | 0.3% | 0.3% |
| Intrasec | 2 | 0.5% | 0.6% |
| Lactamox | 1 | 0.3% | 0.3% |
| Lactigan De Carval | 1 | 0.3% | 0.3% |
| Lactigan Premium | 1 | 0.3% | 0.3% |
| Lactigan Primiun | 1 | 0.3% | 0.3% |
| Lactigan Secado | 1 | 0.3% | 0.3% |
| Mamisan S | 2 | 0.5% | 0.6% |
| Mamyzin Secado | 2 | 0.5% | 0.6% |
| Masticilina Secado O Extrasecado | 27 | 7.4% | 7.6% |
| Mastishot | 2 | 0.5% | 0.6% |
| Mastishot Secado | 1 | 0.3% | 0.3% |
| Mastizur | 1 | 0.3% | 0.3% |
| Naspensal | 1 | 0.3% | 0.3% |
| None | 2 | 0.5% | 0.6% |
| Do not remember | 6 | 1.6% | 1.7% |
| Orbenin | 1 | 0.3% | 0.3% |
| Rilexine | 2 | 0.5% | 0.6% |
| Secador | 1 | 0.3% | 0.3% |
| Secamas | 12 | 3.3% | 3.4% |
| Secamas Secado | 1 | 0.3% | 0.3% |
| Secamas, Bovisec | 1 | 0.3% | 0.3% |
| Secamax | 4 | 1.1% | 1.1% |
| Secamil | 78 | 21.3% | 21.9% |
| NA | 10 | 2.7% | - |

| 67. Which of the following antibiotics do you use most frequently or have you used during the lactation period of animals? (Select only one answer) | | | |
| --- | --- | --- | --- |
| lact_abs | n | percent | valid_percent |
| Amoxicilina | 2 | 0.5% | 0.6% |
| Amoxicilina Y Penicilina | 1 | 0.3% | 0.3% |
| Amoxisol | 1 | 0.3% | 0.3% |
| Anistan | 1 | 0.3% | 0.3% |
| Aspiramicina, Dalipen | 1 | 0.3% | 0.3% |
| Benzetacil | 2 | 0.5% | 0.6% |
| Benzetacil, Tiofur | 1 | 0.3% | 0.3% |
| Betaplusina | 1 | 0.3% | 0.3% |
| Calimas | 1 | 0.3% | 0.3% |
| Calimax | 1 | 0.3% | 0.3% |
| Calimax Lactancia | 3 | 0.8% | 0.8% |
| Cefalesporinas | 1 | 0.3% | 0.3% |
| Cefalexina | 2 | 0.5% | 0.6% |
| Ceftiofur Sodico | 1 | 0.3% | 0.3% |
| Ceftiomastin | 1 | 0.3% | 0.3% |
| Cepravin | 1 | 0.3% | 0.3% |
| Cetiofur | 1 | 0.3% | 0.3% |
| Ciprolac | 1 | 0.3% | 0.3% |
| Clavamox | 7 | 1.9% | 2.0% |
| Clavamox Mastizur Y Mastishot | 2 | 0.5% | 0.6% |
| Clavamox, Mastishot | 1 | 0.3% | 0.3% |
| Clomastin | 1 | 0.3% | 0.3% |
| Clordelin Inyectable Y Cefquiron Inyectable | 1 | 0.3% | 0.3% |
| Cobactan | 58 | 15.8% | 16.3% |
| Cripen L.a. | 1 | 0.3% | 0.3% |
| Flumastin | 1 | 0.3% | 0.3% |
| Ganapen | 3 | 0.8% | 0.8% |
| Ganapen Y Multibio | 1 | 0.3% | 0.3% |
| Gentamicina | 1 | 0.3% | 0.3% |
| Gentax Con Domosyn Y Dextrosa | 1 | 0.3% | 0.3% |
| Kalamu | 1 | 0.3% | 0.3% |
| Kiropen | 1 | 0.3% | 0.3% |
| Kyropen | 2 | 0.5% | 0.6% |
| Lactigan | 2 | 0.5% | 0.6% |
| Lactigan - Ciprolac Lactancia | 1 | 0.3% | 0.3% |
| Lactigan Lactancia | 2 | 0.5% | 0.6% |
| Lactofur | 1 | 0.3% | 0.3% |
| Lactomas, Bicarpen | 1 | 0.3% | 0.3% |
| Latigan Lactancia | 1 | 0.3% | 0.3% |
| Lesporina | 52 | 14.2% | 14.6% |
| Macroespiran De 13millones | 1 | 0.3% | 0.3% |
| Mamin, Ubrolexin, Rilexine | 1 | 0.3% | 0.3% |
| Mamyzin | 25 | 6.8% | 7.0% |
| Mastifin | 1 | 0.3% | 0.3% |
| Mastifin Lactancia | 1 | 0.3% | 0.3% |
| Mastilac | 1 | 0.3% | 0.3% |
| Mastiplan | 2 | 0.5% | 0.6% |
| Mastishot | 69 | 18.9% | 19.4% |
| Mastishot, Cobactan | 1 | 0.3% | 0.3% |
| Mastisur | 3 | 0.8% | 0.8% |
| Mastizur | 15 | 4.1% | 4.2% |
| Mastizur, Neoclordelin | 1 | 0.3% | 0.3% |
| Multivia | 1 | 0.3% | 0.3% |
| Neoclordelin | 6 | 1.6% | 1.7% |
| Neoflordelin | 1 | 0.3% | 0.3% |
| Neomicina | 1 | 0.3% | 0.3% |
| Neucordelin | 1 | 0.3% | 0.3% |
| None | 1 | 0.3% | 0.3% |
| None intramammary in milking period | 1 | 0.3% | 0.3% |
| Do not remember | 19 | 5.2% | 5.3% |
| Penicilina | 2 | 0.5% | 0.6% |
| Secamas Lactancia | 1 | 0.3% | 0.3% |
| Tiofur | 1 | 0.3% | 0.3% |
| All of the above | 1 | 0.3% | 0.3% |
| Trifen | 1 | 0.3% | 0.3% |
| Trimediazina | 1 | 0.3% | 0.3% |
| Trimetropin Sulfa | 1 | 0.3% | 0.3% |
| Tripen | 1 | 0.3% | 0.3% |
| Tripen La | 1 | 0.3% | 0.3% |
| Tylan | 2 | 0.5% | 0.6% |
| Ubrolexin | 5 | 1.4% | 1.4% |
| Ultraclox | 3 | 0.8% | 0.8% |
| Uniclap | 1 | 0.3% | 0.3% |
| Unimas | 1 | 0.3% | 0.3% |
| Urocelin | 1 | 0.3% | 0.3% |
| Other | 1 | 0.3% | 0.3% |
| Vetacristicilina | 1 | 0.3% | 0.3% |
| Vetasiscristicina | 1 | 0.3% | 0.3% |
| Veterflucina | 8 | 2.2% | 2.2% |
| Veterflucina, Multibio, Mastiplan | 1 | 0.3% | 0.3% |
| Veterfluxina | 4 | 1.1% | 1.1% |
| NA | 10 | 2.7% | - |

| 68. Have you attended any course or training on the use of antibiotics? (Select one option only) | | |
| --- | --- | --- |
| train_abs | n | percent |
| Yes | 164 | 44.8% |
| No | 202 | 55.2% |

| 69. Have you heard about antibiotic resistance in the bacteria that cause mastitis? (Select one option only) | | |
| --- | --- | --- |
| amr_know | n | percent |
| Yes | 322 | 88.0% |
| No | 44 | 12.0% |

| 70. If yes, where did you learn about antibiotic resistance? (Select all that apply) | | | |
| --- | --- | --- | --- |
| where_amr_know | n | percent | valid_percent |
| Training | 5 | 1.4% | 1.6% |
| University | 29 | 7.9% | 9.0% |
| Community setting | 5 | 1.4% | 1.6% |
| Internet o Social networks | 10 | 2.7% | 3.1% |
| Internet o Social networks, Informed by the veterinarian | 7 | 1.9% | 2.2% |
| Internet o Social networks, Informed by the veterinarian, Awareness campaign | 6 | 1.6% | 1.9% |
| Informed by the veterinarian | 107 | 29.2% | 33.3% |
| Awareness campaign | 68 | 18.6% | 21.2% |
| Awareness campaign, Informed by the veterinarian | 57 | 15.6% | 17.8% |
| Other | 27 | 7.4% | 8.4% |
| NA | 45 | 12.3% | - |

| 71. Do you think antibiotic resistance is a problem for your farm? (Select only one answer) | | |
| --- | --- | --- |
| amr_prob_farm | n | percent |
| Yes | 181 | 49.5% |
| No | 185 | 50.5% |
